# Sterilizing immunity to influenza virus infection requires local antigen-specific T cell response in the lungs

**DOI:** 10.1038/srep32973

**Published:** 2016-09-06

**Authors:** Avijit Dutta, Ching-Tai Huang, Chun-Yen Lin, Tse-Ching Chen, Yung-Chang Lin, Chia-Shiang Chang, Yueh-Chia He

**Affiliations:** 1 Division of Infectious Diseases, Department of Medicine, Chang Gung Memorial Hospital and Chang Gung University, Taoyuan- 33333, Taiwan; 2 Division of Hepatogastroenterology, Department of Medicine, Chang Gung Memorial Hospital and Chang Gung University, Taoyuan- 33333, Taiwan; 3 Department of Pathology, Chang Gung Memorial Hospital and Chang Gung University, Taoyuan- 33333, Taiwan; 4 Division of Hematology and Oncology, Department of Medicine, Chang Gung Memorial Hospital and Chang Gung University, Taoyuan- 33333, Taiwan

## Abstract

Sterilizing immunity is a unique immune status, which prevents effective virus infection into the host. It is different from the immunity that allows infection but with subsequent successful eradication of the virus. Pre-infection induces sterilizing immunity to homologous influenza virus challenge in ferret. In our antigen-specific experimental system, mice pre-infected with PR8 influenza virus through nasal route are likewise resistant to reinfection of the same strain of virus. The virus is cleared before establishment of effective infection. Intramuscular influenza virus injection confers protection against re-infection with facilitated virus clearance but not sterilizing immunity. Pre-infection and intramuscular injection generates comparable innate immunity and antibody response, but only pre-infection induces virus receptor reduction and efficient antigen-specific T cell response in the lungs. Pre-infection with nH1N1 influenza virus induces virus receptor reduction but not PR8-specific T cell immune response in the lungs and cannot prevent infection of PR8 influenza virus. Pre-infection with PR8 virus induced PR8-specific T cell response in the lungs but cannot prevent infection of nH1N1 virus either. These results reveal that antigen-specific T cell immunity is required for sterilizing immunity.

Influenza viruses cause annual outbreaks and pandemics from time to time and cause significant death every year (http://www.cdc.gov/flu/about/disease/). In surviving people, first encounter with a strain of influenza virus induces an immune response which provides significant protection against subsequent exposure of the same virus strain^1^. The protection perhaps become effective within the span of a flu-season, as successive reinfection by an exactly same strain of influenza virus in a flu-season is quite uncommon^2^. Apart from natural infections, people acquire anti-influenza immunity with vaccination. Intranasal vaccination, similar to natural infection, induces better immunity than intramuscular injection^3^,^4^,^5^,^6^.

Immunity to influenza virus infection involves both the innate and adaptive immune responses. Beyond first line defense with innate immune system, the adaptive immune responses are recruited to limit the amplification and to enhance the clearance of the virus. The adaptive immunity is also very important to provide memory against subsequent infection^7^,^8^,^9^,^10^,^11^. Neutralizing antibodies from B cells is a key component in anti-influenza immunity. Antibodies against viral hemagglutinin (HA) have been extensively studied and always titrated as the level of anti-influenza immunity^11^,^12^,^13^, http://www.who.int/influenza/gisrs_laboratory. T cells also play an important role in anti-influenza immunity, with acute response to first infection and memory response to reinfection. CD4+ T cells provide help for antibody production, and orchestrate cytolytic CD8+ T cell activity and memory T cell generation. CD8+ T cells may support protective immunity even in absence of CD4+ T cell and antibody responses^7^,^8^,^9^,^14^. This significant body of data suggests that, in addition to B cell mediated humoral immunity, antigen-specific T cell response is also very important for effective immunity to influenza virus infection.

Acquired anti-influenza immunity with vaccination is not adequate in general and many vaccinated individuals still suffer from severe disease^15^,^16^,^17^,^18^,^19^,^20^. Although protection varies with how well vaccine strains are matched with the circulating strains, inadequate protection indicates suboptimal immune priming with vaccines^16^,^17^,^18^,^19^,^20^,^21^,^22^. Mechanistic investigation of anti-influenza immunity is desired for further improvement in influenza vaccine development.

In 2010, Laurie *et al*. demonstrated sterilizing immunity to influenza virus infection in ferrets^23^. Pre-infection induced sterilizing immunity which blocked subsequent infection of challenge with homologous strain of virus. They also demonstrated that, intramuscular injection of inactivated influenza viruses could not block subsequent infection although virus shedding was reduced. Laurie *et al*. restricted focus on virus shedding, transmission frequency and survival in ferrets, whereas data reveal a most desired state of protection against re-infection of influenza virus^23^. Sterilizing immunity implies a unique immune status where host immune response blocks effective virus infection as well as the disease.

In this report, we focus on sterilizing immunity through the use of an influenza hemagglutinin (HA) antigen-specific transgenic mouse model. Pre-infection with intranasal inoculation of low-dose live PR8 virus induced sterilizing immunity in mice, which prevented subsequent effective infection of challenge with the same PR8 strain of virus. Mice were first exposed with low-dose live PR8 influenza A virus by either intranasal inoculation or intramuscular injection. They were challenged later with lethal-dose of the same strain of PR8 influenza virus. Mice were protected against lethal challenge with elevated levels of innate immune responses and virus-neutralizing antibodies. However, only intranasal inoculation induced sterilizing immunity. Intramuscular injection did not block subsequent infection but still provide protection with enhanced virus clearance. Intranasal inoculation induced reduction in viral receptors in the lungs which was not seen with intramuscular injection. Receptor reduction decreased susceptibility across different strains of influenza virus, but sterilization only took effect with challenge of the same strain of influenza virus. Intranasal inoculation induced robust local antigen-specific T cell response in the lungs that would not be effective against antigen-mismatched strain of virus. We conclude HA antigen-specific T cell response at the local site of infection is required for sterilization immunity.

## Results

### Pre-infection or intramuscular injection of the virus confers protection against lethal homologous influenza virus challenge with comparable innate and antibody responses

**Figure 1:**
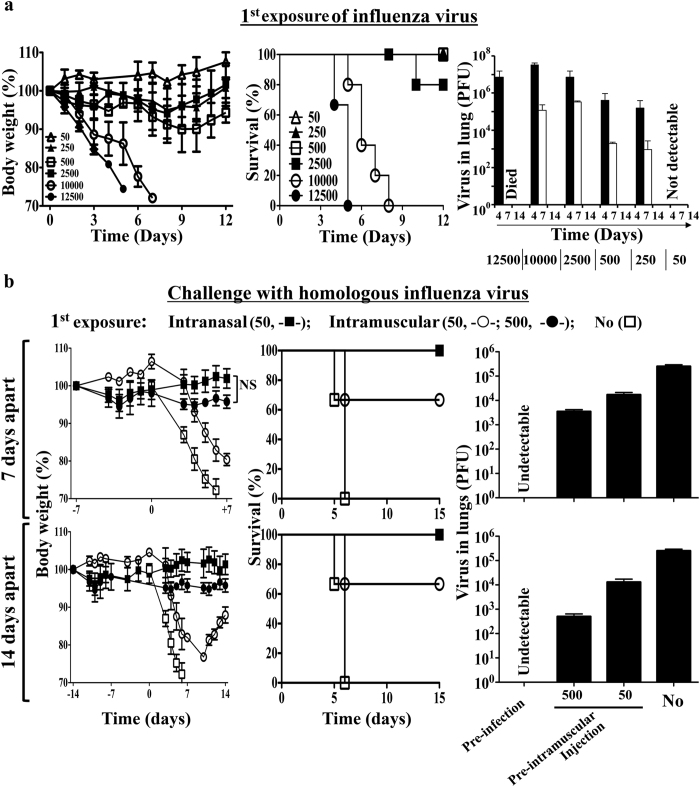
Mice acquire protection against influenza virus infection with pre-infection or prior intramuscular injection of the same virus strain. (**a**) Body weight loss, survival and lung virus titer following infection with stated doses of PR8 influenza virus through intranasal inoculation in naïve mice. (**b**) Body weight loss, survival and lung virus titer following challenge of 1.25 × 10^4 ^PFU of PR8 influenza virus in mice that received stated doses of PR8 virus through intranasal inoculation (pre-infection) or intramuscular injection 7- or 14- days ago. Virus titers in the lungs were measured by plaque assay on day 6- post infection/challenge. Data are representative of at least three similar experiments and presented as mean ± s.d. (n = 6/group).

Naïve BALB/c mice are susceptible to influenza virus infection. Intranasal inoculation of 1.0~1.25 × 10^4^ plaque-forming units (PFU) of live PR8 strain H1N1 influenza virus caused disease with ruffled hair and body weight loss from day 2- post infection. They succumbed to death between day- 5 to day- 8. Disease symptoms were delayed with decreased doses of infection. 0.25~2.5 × 10^3 ^PFU caused ruffled hair and body weight loss only after day 4- post infection. The majority recovered from the disease since day 7- post infection and survived. The virus titers in the lungs reached the peak on day 4- post infection and declined thereafter as analyzed by plaque assay. The virus was eventually cleared from the lungs of mice that survived on day 14- post infection. Further decrease of intranasal inoculation dose to 50 PFU caused no significant illness and the virus can never be detected in the lungs (Fig. 1a). Intramuscular injection of 50~500 PFU of live virus caused no illness either (‘Day -7 to 0’ and ‘Day -14 to 0’ of Fig. 1b) and the virus was never be detected in the lungs as well.

Mice may survive lethal challenge of 1.25 × 10^4 ^PFU PR8 virus if they had been exposed to the PR8 virus. Intranasal inoculation of 50 PFU or intramuscular injection of 500 PFU conferred protection with very little loss of weight. Intramuscular injection of 50 PFU conferred protection but the body loss was significant. Virus cannot be detected in the lungs after lethal challenge with protection by intranasal inoculation. Intramuscular injection accelerated the virus clearance from the lungs with lethal challenge (Fig. 1b).

**Figure 2:**
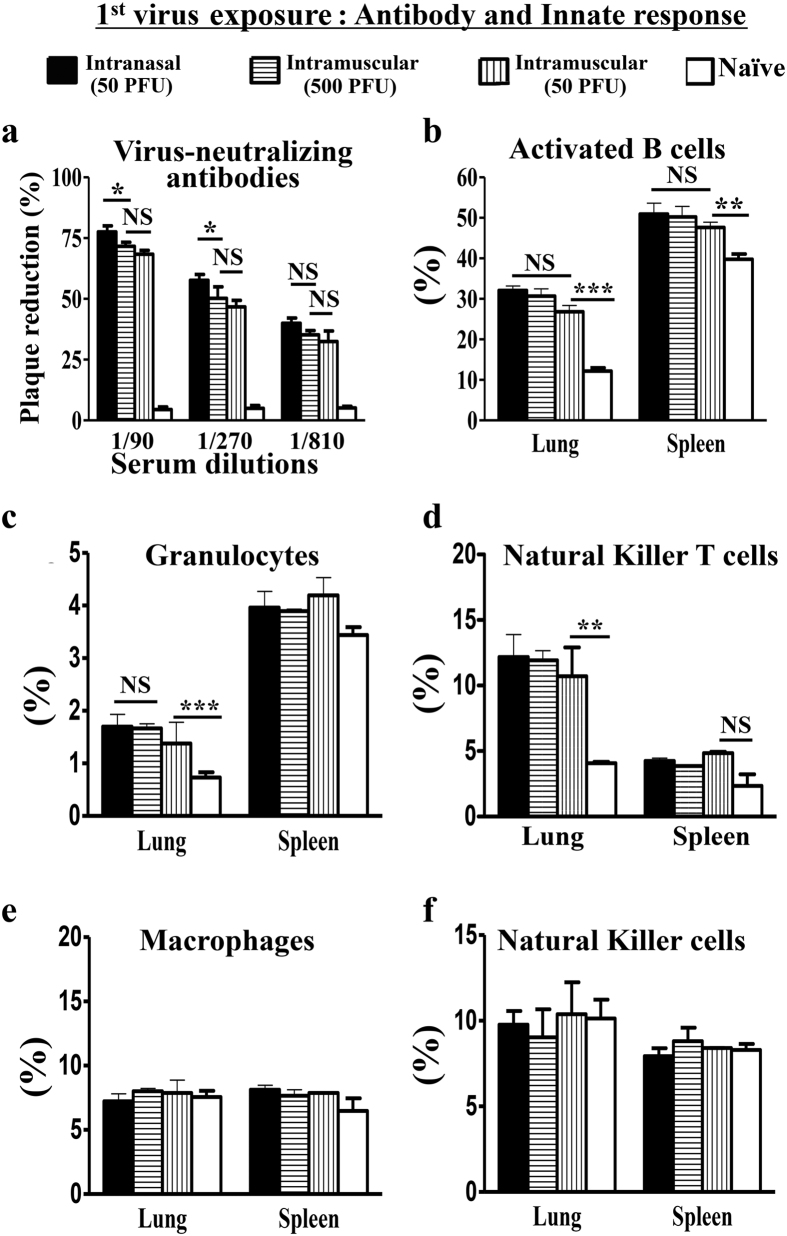
Pre-infection and intramuscular influenza virus injections induce comparable antibody response and innate immunity. Naïve mice received intranasal inoculation (pre-infection) or intramuscular injection of stated doses of PR8 influenza virus. 7-days apart, Sera or stated organs were analyzed. Naïve mice without virus exposure served as controls. Data are representative of at least three similar experiments and presented as mean ± s.d. (***p < 0.0001; **p < 0.001; *p < 0.01; NS = non-significant, p > 0.05; n = 6/group).

Intranasal inoculation of 50 PFU or intramuscular injection of 50~500 PFU of PR8 virus induced neutralizing antibody response against PR8 virus, as measured by *in vitro* plaque reduction assay. Virus neutralization by the antibodies was comparable between intranasal inoculation and intramuscular injection, both in the sera and in the lung lysates. There were also increases of activated B cells (B220+CD38+), neutrophils (Gr1+), and natural killer T cells (CD3+NK1.1+) in the lungs and spleens. The levels between intranasal inoculation and intramuscular injection were quite similar, too (Fig. 2b–d). There was no increase in F4/80+ macrophages and NK1.1+ natural killer cells in the lungs and the spleens (Fig. 2e,f). There was no increase of IFN-α in the sera and the lung lysates, as measured by ELISA. Taken together, both intranasal inoculation and intramuscular injection of the virus induces comparable functional antibody and innate immune responses.

### Pre-infection with intranasal inoculation induces sterilization immunity

**Figure 3:**
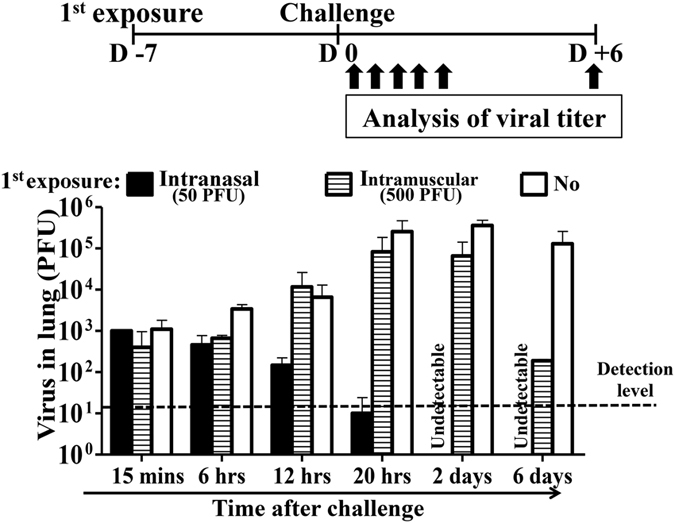
Limited amplification of challenge virus in the lungs of pre-infected mice. Naïve mice received pre-infection (intranasal inoculation) or intramuscular injection of stated doses of PR8 influenza virus. 7-days apart, mice were challenged with 1.25 × 10^4^ PFU PR8 virus. Naïve mice with challenge only, served as controls. Virus titers in the lungs were measured by plaque assay on stated time points after the challenge. Data are representative of at least three similar experiments and presented as mean ± s.d. (n = 6/group).

We studied the viral titer kinetics in the lungs of mice following 1.25 × 10^4 ^PFU lethal challenge, with protection from either 500 PFU intramuscular injection or pre-infection with 50 PFU intranasal inoculation. Virus reached the lungs within 15 minutes of lethal challenge in mice with either intranasal or intramuscular route of protection. There was a rapid decline of viral titers between 6 to 20 hours in mice with protection from intranasal inoculation. The virus was no more detectable by day 2- post challenge (Fig. 3). Virus replicated and the titers increased between 12 hours and 2 days in mice with protection from intramuscular injection. The kinetics was similar in mice with protection from intramuscular injection and in control mice with no protection. However, clearance of the virus was accelerated in mice with protection from intramuscular injection. The viral titer was significantly lower than control mice on day 6 after lethal challenge (Fig. 3).

**Figure 4:**
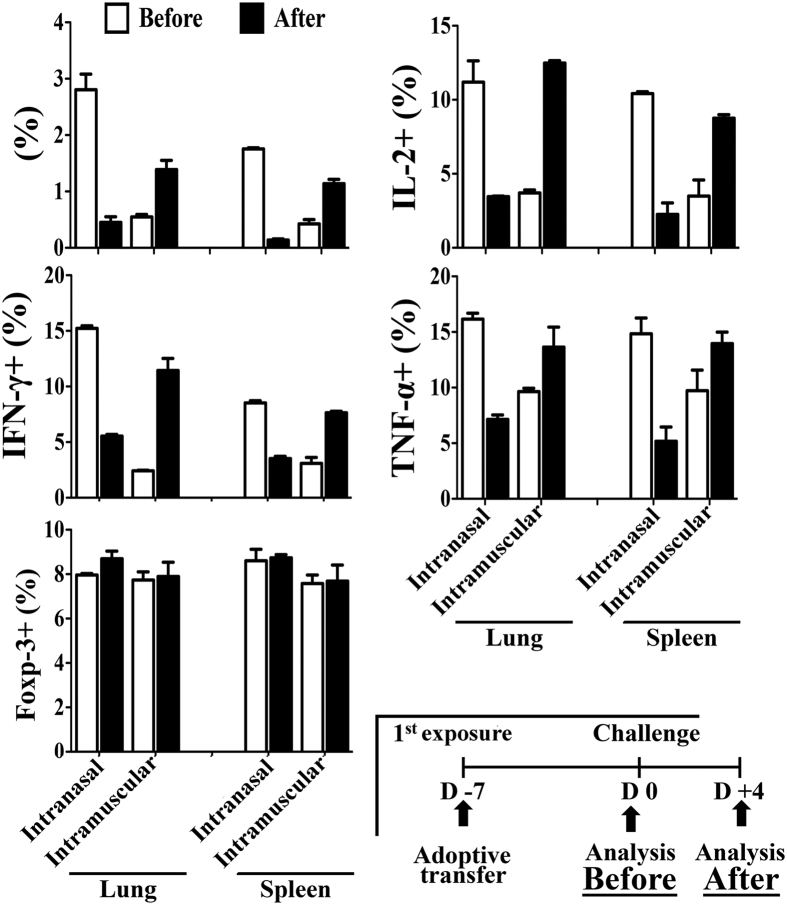
No further activation of HA-specific CD4+ T cells in response to challenge virus in pre-infected mice. Mice received 2.5 × 10^6^ naïve HA-specific CD4+ T cells on the same day with pre-infection (50 PFU, intranasal inoculation) or intramuscular injection (500 PFU) of PR8 virus. On day 7-post virus exposure, half of the mice were sacrificed to study activation status of HA-specific CD4+ T cells (Before) and rest of the mice were challenged with 1.25 × 10^4^ PFU of PR8 virus. On day 4- post challenge, mice were sacrificed to study activation status of HA-specific CD4+ T cells similarly (After). Data are representative of at least three similar experiments and presented as mean ± s.d. (n = 6/group).

Disease manifestations and virus load kinetics implied that the virus of lethal challenge did not amplify and eventually cleared without establishment of effective infection in mice with previous intranasal inoculation (pre-infection). It is also possible that virus of the lethal challenge established mild infection and get cleared by immune responses induced by the pre-infection. As there should be virus antigen shedding with the second possibility, we tested virus antigen shedding by the viruses of lethal challenge in pre-infected mice through antigen-driven activation of influenza hemagglutinin (HA) antigen-specific 6.5 CD4+ T cells. At first, naïve HA-specific 6.5 CD4+ T cells were adoptively transferred on the same day with first virus exposure^24^,^25^. Virus antigens of the first intranasal inoculation (pre-infection) activated adoptively transferred naïve HA-specific 6.5 CD4+ T cells on day 7 after pre-infection (Before, Fig. 4). The activation was not augmented by the virus antigens of lethal challenge as revealed by analysis of activation status on day 4- post challenge (After, Fig. 4). In fact, clonotypic percentage and production of cytokines such as IFNγ, IL-2 and TNF-α was actually reduced after the lethal challenge (Fig. 4). Similar very weak T cell recall response has been reported earlier too^26^. In our experiments, the absence of additional activation by the lethal challenge was not due to suppression by HA-specific regulatory T cells (Treg). The percentage of HA-specific Treg (6.5+CD4+CD25+Foxp3+) was not increased after the challenge (Fig. 4). In contrast, the virus antigens of lethal challenge resulted in significant additional activation of HA-specific 6.5 CD4+ T cells, which had been activated by prior intramuscular injection, with more clonotypic expansion and production of effector cytokines such as IFNγ, IL-2 and TNF-α (Fig. 4).

**Figure 5:**
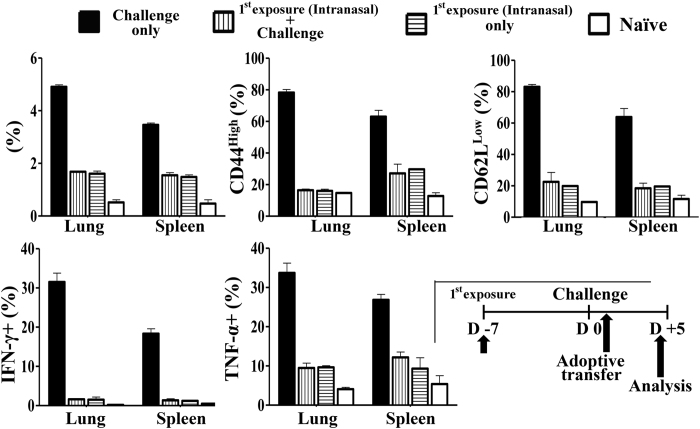
No activation of naïve HA-specific CD4+ T cells in response to challenge virus in pre-infected mice. Naïve mice received pre-infection (intranasal inoculation) of 50 PFU PR8 virus and challenge of 1.25 × 10^4 ^PFU PR8 virus 7 days apart. Naïve 2.5 × 10^6^ HA-specific CD4+ T cells were adoptively transferred in mice on the next day of challenge. Mice with pre-infection but no challenge received similar cell transfer and served as controls. Another group of naïve mice also received same dose virus challenge and equal number cell transfer, serving as another control group. Data are representative of at least three similar experiments and presented as mean ± s.d. (n = 6/group).

We then tested whether the virus antigens of lethal challenge could activate naïve 6.5 HA-specific T cells in mice with protection from prior 50 PFU intranasal inoculation. Naïve 6.5 HA-specific CD4+ T cells were adoptively transferred one day after the lethal challenge. Naïve 6.5 HA-specific CD4+ T cells were not activated by the virus antigens of lethal challenge, as compared to the 6.5 cells adoptively transferred into control mice with the same 50 PFU intranasal inoculation but no subsequent lethal challenge. The clonotypic percentage, CD44 up-regulation, CD62L down-regulation, and the production of effector cytokines IFNγ and TNF-α were comparable on day 4- post adoptive transfer (Fig. 5). Naïve mice with synchronized lethal challenge without prior viral exposure served as another group of control. 6.5 HA-specific CD4+ T cells were robustly activated by the lethal challenge (Fig. 5). Taken together, lethal challenge did not provide virus antigens for HA-specific CD4+ T cell activation in mice with protection from prior intranasal inoculation (pre-infection). These results imply that pre-infection induces sterilizing immunity which prevents viruses of lethal challenge to amplify and the viruses of lethal challenge are cleared without shedding of significant antigen and establishment of effective infection.

### Pre-infection with intranasal inoculation reduces virus receptors in the lungs

**Figure 6:**
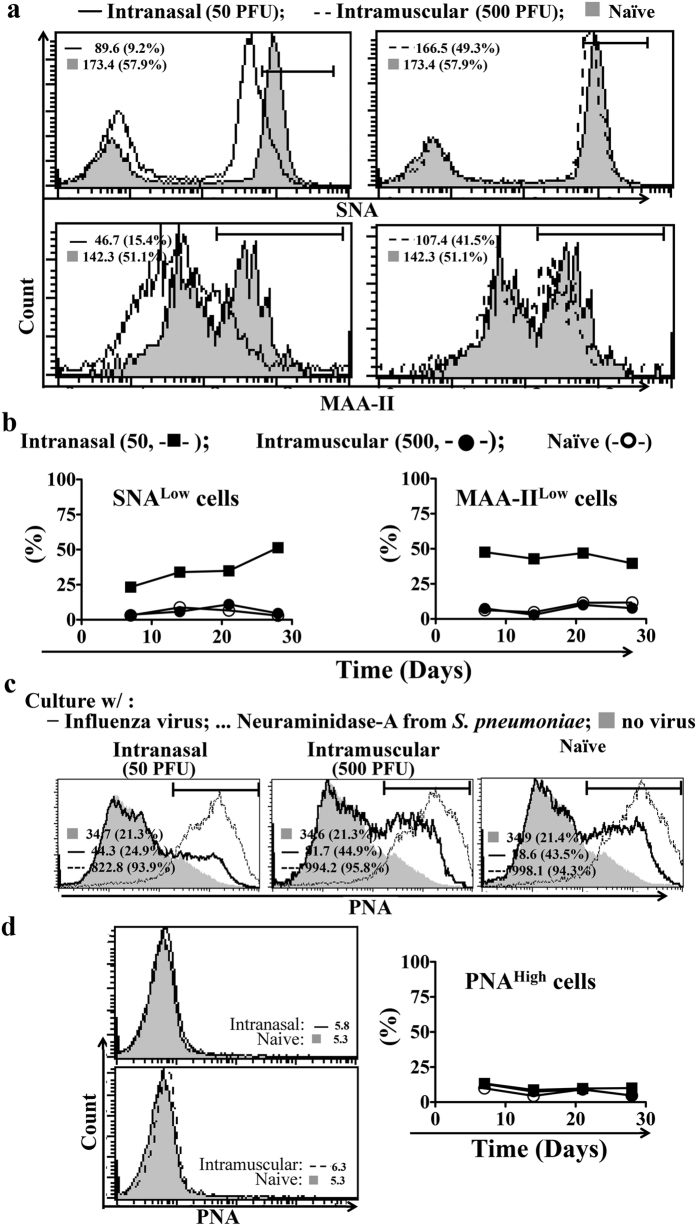
Pre-infection reduces viral receptors on lung cells causing the cells less susceptible to subsequent virus exposure. (**a**) Levels of virus receptors with α-2,6 (SNA bound) and α-2,3 (MAA-II bound) sialic acid linkages on lung cells in mice on day 7-post pre-infection (intranasal inoculation) or intramuscular injection of PR8 virus. Naïve mice served as controls. (**b**) The percentage of lung cells with decreased α-2,6 and α-2,3 sialic acids during the stated period after virus inoculation. Naïve mice served as controls. (**c**) Mice with pre-infection or intramuscular injection of the PR8 virus were sacrificed on day 7-post virus exposure. Lung cells were cultured with PR8 influenza virus (0.1 multiplicity of infection) for overnight *in vitro* and desialylation was measured by the binding of lectin PNA. Lung cells of naïve mice served as controls. Neuraminidase-A from *S*. *pneumoniae* was used as a positive control of desialylating agent. (**d**) The percentage of desialylated (PNA^High^) lung cells during the stated period after virus inoculation. Lung cells from naïve mice served as controls. Data are representative of at least three similar experiments and presented as mean ± s.d. (n = 6/group).

Receptors with terminal α-2,3- and α-2,6- linked sialic acid residues on host cell surface are important for influenza virus infection^27^. Influenza virus attaches to host cells in the lungs with the biding of hemagglutinin (HA) on these receptors and enters the cell with neuraminidase (NA) mediated cleavage of α-2,3 and α-2,6 sialic acid residues^28^,^29^. Intranasal inoculation of 50 PFU PR8 influenza virus reduced the level of α-2,3 and α-2,6 sialic acids on lung cell surface, as revealed by reduced binding of α-2,3 sialic acid ligand Maackia amurensis lectin (MAA-II) and α-2,6 sialic acid ligand Sambucus nigra lectin (SNA) on day 7- post inoculation (Fig. 6a). Intramuscular injection of the virus did not result in reduction, with MAA-II and SNA binding comparable to that of non-infected mice (Fig. 6a). The reduced levels of receptors with α-2,3 and α-2,6 sialic acid following intranasal inoculation was maintained up to at least 28 days after intranasal inoculation (Fig. 6b).

The change in receptors with intranasal inoculation may result in decreased viral attachment and decreased NA-mediated cleavage of α-2,3 and α-2,6 sialic acid residues up on viral entry with further challenge with influenza virus. We tested NA-mediated cleavage of the sialic acids (desialylation) through peanut agglutinin lectin (PNA) staining. Sialic acids cover PNA binding motifs with surface galactosyl residues, including Gal-β(1-3)-GalNAc and PNA staining will be negative in the presence of un-cleaved sialic acids. There was minimal desialylation of lung cells from mice with prior intranasal inoculation in overnight culture with PR8 virus, as revealed by negative PNA staining (Fig. 6c). The desialylation was significant on lung cells from mice with prior intramuscular injection or naïve non-infected mice, as revealed by positive PNA staining (Fig. 6c). Taken together, virus receptors were reduced on the lung cells of intranasally virus-inoculated mice and these lung cells were less susceptible to further infection of influenza virus.

Prior to further exposure of PR8 virus *in vitro*, PNA staining was negative for lung cells of mice with intranasal inoculation or intramuscular injection as well as of the naïve healthy mice (Fig. 6c,d). Negative PNA staining implied *de novo* sialic acid synthesis re-sialylated lung cells in a continuous process *in vivo*, although a decreased share of α-2,3 and α-2,6 sialic acids was persisted on the lung cell surface in mice with intranasal inoculation.

### Pre-infection with intranasal inoculation recruits efficient on-site antigen-specific T cell response in the lungs

**Figure 7:**
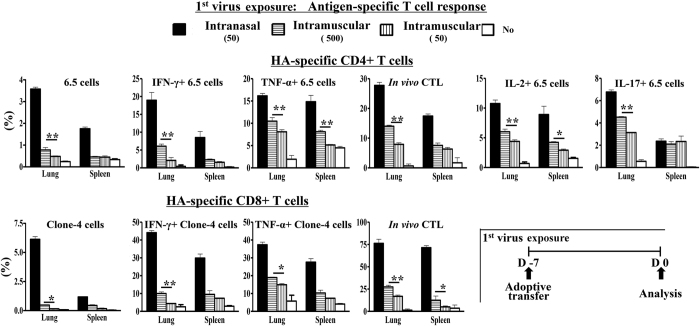
Pre-infection induces efficient antigen-specific T cell activation in the lungs. Activation status of adoptively transferred HA-specific T cells in terms of their clonotypic percentage, production of stated cytokines and *in vivo* cytolytic activity in the lungs and spleens of mice with pre-infection (intranasal inoculation, 50 PFU) or intramuscular injection (500 PFU) of PR8 virus on day 7 post adoptive transfer and virus exposure. Naïve mice received similar adoptive transfer of 2.5 × 10^6^ naïve HA-specific CD4+ or CD8+ T cells without virus exposure and served as controls. Data are representative of at least three similar experiments and presented as mean ± s.d. (**p < 0.001; *p < 0.01; n = 6/group).

Naïve HA-specific 6.5 CD4+ or Clone-4 CD8+ T cells were adoptively transferred into recipient mice on the same day with intranasal inoculation or intramuscular injection of PR8 influenza virus. HA-specific CD4+ or CD8+ T cell responses were compared among different groups of mice on day 7. Intranasal inoculation of 50 PFU PR8 virus activated CD4+ or CD8+ HA-specific T cells in the lungs with significant clonotypic expansion, production of effector cytokines, such as IL-2, IFN-γ, TNF-α and IL-17a and *in vivo* cytolytic activity (Fig. 7). Intramuscular injection of 50 PFU PR8 virus barely activated any of the CD4+ or CD8+ T cells in the lungs (Fig. 7). Intramuscular injection of 500 PFU virus recruited activated HA-specific CD4+ or CD8+ T cells in the lungs as well, but the activation status of these HA-specific CD4+ or CD8+ T cells was significantly inferior than that of 50 PFU intranasal inoculation, with less clonotypic expansion, less production of effector cytokines such as IL-2, IFN-γ, TNF-α and IL-17a, and lower *in vivo* cytolytic activity (Fig. 7). Local antigen-specific CD4+ or CD8+ T cell responses in the lungs induced by intranasal inoculation was more efficient than that recruited by intramuscular injection.

### Sterilizing immunity requires efficient local antigen-specific T cell responses in the lungs

**Figure 8:**
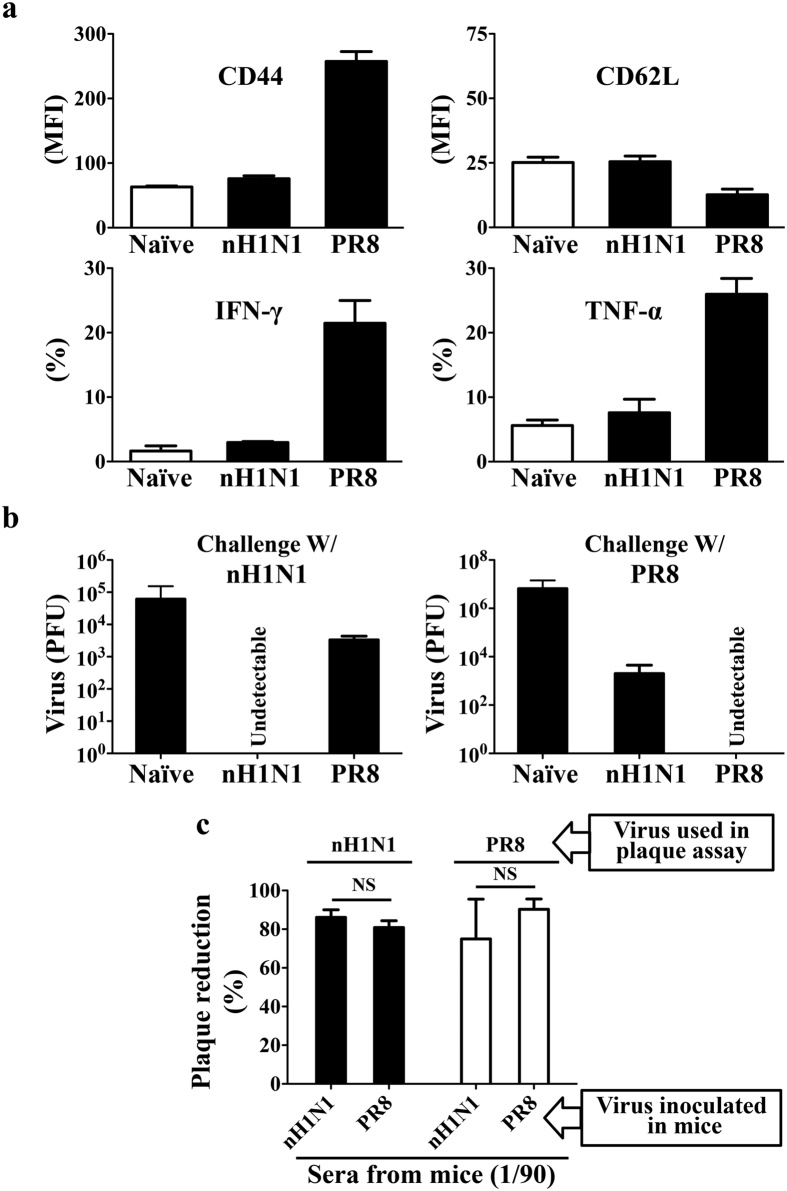
Sterilizing immunity requires local antigen-specific T cell response in the lungs. (**a**) HA-specific 6.5 CD4+ T cell response to the 50 PFU intranasal inoculation of antigen-mismatched nH1N1 or antigen-matched PR8 virus in the lungs of mice on day 7-post adoptive transfer and virus inoculation. Naïve mice received similar adoptive transfer of 2.5 × 10^6^ naïve HA-specific 6.5 CD4+ T cells without virus exposure and served as controls. (**b**) Lung virus titer on day 2-post challenge of heterologous and homologous virus on day 7-after pre-infection in mice pre-infected with stated virus strains. Mice with challenge but no pre-infection (naive) served as controls. (**c**) Neutralization of *in vitro* influenza virus infection in MDCK cells by the sera from mice pre-infected with stated strains of influenza virus on day 7. Sera from naive mice without virus inoculation served as naïve controls. Data are representative of at least three similar experiments and presented as mean ± s.d. (NS = p > 0.05, non significant; n = 6/group).

We demonstrated reduction of virus receptors and generation of effective local antigen-specific T cell responses in the lungs with intranasal inoculation (pre-infection) induced sterilizing immunity. We further tested the relative importance of antigen-specific T cell responses in sterilizing immunity with different combination of influenza virus strains for intranasal inoculation and subsequent challenge. Sterilization immunity was effective if virus strains were the same for both intranasal inoculation and challenge. Intranasal inoculation with 50 PFU PR8 influenza virus induced sterilizing immunity against challenge with PR8 virus and intranasal inoculation with 50 PFU Novel H1N1 (nH1N1) influenza virus induced sterilizing immunity against challenge with nH1N1 virus (Fig. 8a). Intranasal inoculation with 50 PFU of nH1N1 influenza virus induced receptor reduction but not PR8 influenza virus HA-specific 6.5 CD4+ T cell response in the lungs. CD44 up-regulation, CD62L down-regulation and production of effector cytokines IFN-γ and TNF-α of the 6.5 CD4+ T cells on day 7- post inoculation were comparable to that of cells in non-infected mice cells (Fig. 8b). The nH1N1 intranasal inoculation did not result in sterilization immunity to challenge with PR8 virus. There was a significant load of PR8 virus in the lungs on day 2- post the lethal challenge (Fig. 8b). Intranasal inoculation with 50 PFU PR8 virus induced receptor reduction as well as PR8 influenza virus HA-specific 6.5 CD4+ T cell response in the lungs. The PR8 intranasal inoculation did not result in sterilization immunity to challenge with nH1N1 virus either (Fig. 8a,b).

We also tested the antibody responses in these combinations of intranasal inoculation and subsequent challenge with plaque reduction assay. On day 7- post intranasal inoculation of either PR8 or nH1N1 strains of influenza virus, there were neutralizing antibodies in de-complemented sera which were cross-reactive to each other. Sera from nH1N1 virus inoculated mice neutralized both nH1N1 and PR8 virus infection *in vitro*. Sera from PR8 virus inoculated mice neutralized both PR8 and nH1N1 virus infection *in vitro* as well. Antibody levels were comparable as revealed by the comparable neutralization effects in *in vitro* plaque reduction assay (Fig. 8c). Taken together, effective local antigen-specific CD4+ T cell response in the lungs is a critical component of sterilizing immunity in influenza virus infection.

## Discussion

Our results demonstrate sterilizing immunity against homologous influenza virus in mice, similar to previous finding of sterilizing immunity in ferrets^23^. Pre-infection with intranasal inoculation but not intramuscular injection induced sterilization immunity to subsequent challenge of same virus strain. It could be possible that the virus replicated and delivered a higher bolus antigen when administered intranasally compared to that when administered intramuscularly. A higher bolus, with a 10 times higher dose, for intramuscular injection improved the level of protection but failed to induce sterilization effect, suggesting intranasal delivery as a major contributing factor. Intranasal inoculation induced viral neuraminidase-mediated α-2,3 and α-2,6 sialic acid cleavage and recruited efficient local antigen-specific T cell response in the lungs. Reduction of virus receptors with terminal α-2,3 and α-2,6 sialic acid cleavage decreased the susceptibility of lung cells to succeeding influenza virus infection. However, virus receptor reduction was not sufficient for sterilization effect without local antigen-specific T cell response in the lungs. Subsequent challenge of heterologous strain of influenza virus still established effective infection. All these results imply that antigen-specific T cell response is a critical component of anti-influenza immunity as the local response at site of infection is indispensible for desirable sterilization immunity.

Viral neuraminidase mediated α-2,3 and α-2,6 sialic acid cleavage reduced virus receptors on lung cell surface and render them less susceptible to further exposure of influenza virus. Sialic acid down-regulation we demonstrated contributes to the overall protective effect, in addition to the contributions of humoral, cellular and other components of immunity in our experiments. Lower levels of α-2,3- and α-2,6- linked sialic acids on influenza virus infected cells have been reported to be preventive for subsequent infection^30^,^31^. With higher NA content, live-attenuated influenza vaccines (LAIV) provide better protection than inactivated influenza vaccines (TIV).

We demonstrate sterilizing immunity with efficient HA-specific CD4+ and CD8+ T cell response in the lungs with production of IL-2, IFN-γ and TNF-α and cytotoxic activity *in vivo*. IL-2 and IFN-γ are known to support influenza-specific B cells^32^,^33^,^34^ and cytotoxic T cells^35^,^36^,^37^. Cytotoxic T cells, with secretion of perforin, granzyme and IFN-γ, help limit viral replication at the site of infection^36^. However, we revealed that T cell response in the lungs is most effective against influenza virus of matched antigen only. Both PR8 and nH1N1 are of H1N1 strains of influenza virus. They have matched core but not surface HA proteins. There is a recognizable sequence difference in HA protein between these two strains. PR8 HA-specific T cells responded to pre-infection with PR8 virus. This response was associated with sterilization effect against challenge of PR8 virus but not against that of nH1N1 virus.

Among the immune correlates of influenza protection, influenza hemagglutinin (HA) specific neutralization antibody is the most recognized^11^,^12^,^38^, http://www.who.int/influenza. Hemagglutination inhibition (HAI) assay titer greater or equal to 40 is considered as the standard of protection since 1972 (ref. 13). This measurement has been applied for licensure of inactivated human seasonal influenza vaccines (TIV). However, the possibility of vaccine ineffectiveness always prompts the need to understand other relevant protection factors^39^,^40^. In our experiments, both pre-infection with intranasal inoculation and intramuscular injection induced comparable levels of neutralizing antibodies and neutralization antibodies are associated with accelerated virus clearance and protection. Neutralizing antibodies induced by one H1N1 influenza virus strain was cross-reactive to another H1N1 influenza virus strain. However, sterilization effect was only induced by intranasal inoculation and was against the same strain of virus. These results suggest that neutralization antibodies are important for protection, but they are not the sole component of anti-influenza immunity. Protection immunity against influenza virus should be the summation effects of many arms of the immune system. Antigen-specific T cell response plays a role in this consortium as well. It is also very important, as it is the indispensible component of sterilization immunity.

Sterilizing immunity is the most desired immune status that prevents subsequent effective infection of an antigen-matched virus and associated disease. We found that first infection of influenza virus confers sterilizing immunity to subsequent challenge of homologous virus. Establishment of effective infection was prevented with no amplification of the challenge virus. The challenge virus failed to shed antigens sufficient to induce antigen-specific T cell response as well. Naïve cognate antigen specific CD4+ T cells did not respond. This observation is very similar to a previous report that there was only very weak CD4+ T cell recall response to the second challenge of influenza virus^26^. It is possible that they may also reveal blockade of re-infection if they extended their focus beyond characterization of responding T cells and tested the viral load in the lungs.

The effectiveness of flu vaccines is still not remarkably impressive in humans till date^16^,^18^,^20^. While there has been an increase in the number of clinical studies aimed at determining the efficacy and/or effectiveness of current vaccines, there has not been a corresponding increase in the number of laboratory-based studies to determine what underpins these measures. Neutralization antibody and cytotoxic T cell response have been postulated for decades as the major components of influenza immunity, validated by experiments in mouse models and in humans^39^,^41^,^42^,^43^. We revealed two more critical factors for influenza immunity with our study here. Intranasal inoculation resulted in neuraminidase-mediated desialylation and reduced viral receptors. Reduced viral receptors are associated with less susceptibility and potentially can attenuate the magnitude of infection. Intranasal inoculation also instigates on-site antigen-specific T cell activation in the lungs, which is fundamentally required for complete blockade of re-infection of the same strain of influenza virus. Sterilizing immunity is a desired status of infection blockade, which totally precludes disease. Sterilizing immunity is the ultimate goal of influenza vaccination^23^,^44^,^45^. Intramuscular delivery of inactivated virus (TIV) is the most common practice for flu vaccination in whole world. The effectiveness of TIV is not adequate and several vaccinated people suffer from severe disease^4^,^16^,^18^,^20^,^22^,^46^. Improved efficacy with intramuscular injection of live virus^45^ or intranasal delivery of attenuated live virus^47^, http://www.cdc.gov/flu/about/qa/nasalspray.htm suggests live virus in preparation and delivery through natural route provide better protection. Our mechanistic dissection for sterilizing immunity corroborates the critical importance of natural route live virus infection in protection immunity. While neuraminidase activity induced receptor reduction and decreased susceptibility, local antigen-specific T cell response at site of infection optimized anti-influenza immunity with sterilizing effect. Similarly nasal spray vaccines of live attenuated influenza virus in humans confer better protective efficacy with enhanced antigen-specific T cell response in the airway^48^,^49^,^50^.

It is difficult to study the effectiveness of a newly developed flu vaccine in humans with real challenge of influenza virus in vaccinated humans. Translational research with experimental animal models is necessary. However, there are still barriers to the study of adaptive T cell immunity in animal models. The precursor frequency of T cells with any antigen specificity is very low. With the adoptive transfer of HA antigen-specific CD4+ and CD8+ T cells, experiments in our transgenic mouse model revealed that antigen-specific T cell response elevates the level of protection with sterilization effect from the level with accelerated virus clearance only. Although the physiological significance is a concern with unusually high precursor frequency of T cells of certain antigen specificity in adoptive transfer approach, we and others have demonstrated that the frequency of specific T cells with adoptive transfer is not appreciably different from those with physiological T cell expansion^24^,^25^,^51^,^52^,^53^. We also have identified LAG-3 as the target for cancer immunotherapy with this approach and anti-LAG-3 has been in phase I clinical trial since 2013 (ref. 53).

The idea of universal flu vaccine with preparations for immunity against core and conserved structure of virus is very attractive^40^. It would be effective against many different strains of influenza virus. Influenza vaccine strains are selected each year with presumed best match to the circulating strains in the next influenza season (http://www.cdc.gov/flu/about/season/vaccine-selection.htm). Universal vaccine may save this yearly effort and make our community ready for seasonal influenza challenge with strain variation from year to year. However, the core and conserved structures are less immunogenic in general. Immunity against core or conserved component rarely offers adequate protection for strains of different surface protein antigen. In our experiments, priming with PR8 virus did not mobilize sterilization effect against nH1N1 strain although both of them are H1N1 influenza A viruses with minor difference in HA only. Epitope-specific T cell response is essential for sterilization immunity. This argues against the idea of universal flu vaccine. Our results demonstrate the importance of T cell response in influenza immunity and optimized T cell response is a rational goal for vaccine design with better efficacy.

## Methods

### Mice

All mice are of BALB/c background. The TCR-transgenic mouse lines 6.5 expresseing a TCR recognizing an I-E^d^-restricted HA epitope (^110^SFERFEIFPKE^120^) and Clone-4 expresseing a TCR recognizing a K^d^-restricted HA epitope (^518^IYSTVASSL^526^) are on the Thy-1.1/1.1 background. All the mice were kept in individually ventilated cages within specific pathogen-free environment and used for experiments between the ages of 8–24 weeks. All experiments involving the use of mice were performed in accordance with protocols approved by the “Animal Care and Use Committee” of the Chang Gung Memorial Hospital (Approval No. 2008121705).

### First virus exposure and subsequent lethal challenge

For first virus exposure, indicated inoculums of live PR8 (A/PR/8/34) strain of H1N1 influenza A virus in 50 μl of PBS were inoculated intranasally or injected through thigh muscle under light anesthesia^24^,^25^. Inoculum of 1.25 × 10^4 ^PFU, also in 50 μl of PBS, under light anesthesia was used for the lethal challenge. A clinical isolate of novel H1N1 influenza in 2009 (Accession # 09-1768201104060CGMH) was used as heterologous strain of influenza virus for challenge. The mice with severe illness were humanely euthanized if they lost 30% of their body weight, could not reach for food and drink from a readily available source, or were unable to respond to a pinch in the hind leg.

### Estimation of lung virus titer

We measured live virus in organs of influenza virus infected mice using a modified Madin Darby canine kidney cell (MDCK; The American Type Culture Collection) plaque assay^24^,^25^. Organs are collected at the indicated times in 1 ml DMEM, snap frozen in liquid nitrogen and stored at −80 °C until they are ready for use. MDCK monolayers are grown in DMEM supplemented with 10% FCS and Antibiotic-Antimycotic (100 IU/ml of penicillin, 100 μg/ml streptomycin, and 0.25 μg/ml amphotericin B; GIBCO BRL). 10-fold dilutions of the tissue homogenates are prepared in DMEM supplemented with 0.00025% trypsin. 500 μl of each dilution is added to confluent monolayers of MDCK cells in 6 well plates in duplicates for 1 h incubation at 35 °C, 5% CO_2_. Each well receives 2 ml of an agar overlay (0.3%) in medium containing DMEM with 0.00025% trypsin. Cells are incubated for 3 more days at 35 °C and are fixed with 10% formalin for 20 min. The agar overlay is then removed and fixed monolayers are stained by adding 10 fold dilution of 2% crystal violet prepared in 20% ethanol. The results are presented as plaque forming unit (PFU)/ml = (mean number of plaques × 2) × (1/dilution factor).

### Neutralizing antibody level in sera and lung lysates

The functional neutralizing antibody against influenza was quantified by plaque reduction assay as described in our previous publication^24^,^25^. In brief, sera or lung lysates from mice are collected on day 7- post vaccination. The sera or lung lysates are heat inactivated for 30 minutes at 56 °C to de-complement. 50 PFU of live virus was allowed to infect monolayers of DMEM cells in the presence or absence of de-complemented sera with specified dilutions for one hour at 35 °C. Motility of virus was blocked with the use of agarose. Reduction of the plaques due to inhibition by anti-influenza antibody in the sera or lung lysates was counted manually after 72 hours of incubation at 35 °C.

### Analysis of interferon-α response in the sera and lung lysates

Pre-infected or pre-intramuscularly virus injected mice were sacrificed on day 7 after virus exposure. Sera were collected and lungs were homogenized and frozen in −80 °C until they are ready for use. The sera and lung lysates were analyzed for interferon-α through VeriKine™ Mouse Interferon Alpha ELISA Kit (https://www.pblassaysci.com/content/verikine-mouse-interferon-alpha-elisa-kit) as per manufacturer’s instruction.

### Adoptive transfer

Clonotypic TCR transgenic T cells were prepared from pooled spleen and lymph nodes of 6.5 or Clone-4 transgenic mice, and enriched with MACS isolation kits. Clonotypic percentage is determined by flow cytometry analysis. The activation markers CD44 and CD62L were analyzed to ensure that these clonotypic cells are naïve in phenotype. Clonotypic cells (2.5 × 10^6^) were re-suspended in 0.2 ml of HBSS for tail vein injection in recipient mice^24^,^25^.

### Flow cytometry and intracellular cytokine staining

Donor CD4+ or CD8+ T cells were identified using fluorochrome labeled anti-CD4/CD8 and anti-Thy1.1 antibodies. Single cell suspensions were re-stimulated with cognate peptides for 5 hours in the presence of 5 μg/ml brefeldin A. Re-stimulated cells were surface stained, fixed in IC fixation buffer, washed and stained in permeabilization buffer against target cytokines. FACS acquisitions were performed using FACSCalibur (BD Biosciences) and analyses were done with the help of Cellquest Pro (BD Biosciences) or FlowJo (Tree Star, Inc.) software.

### *In vivo* cytotoxicity assay

Syngeneic spleen cells, from wild type BALB/c mice, were used as *in vivo* target cells. They were labeled by incubation for 15 min at 37 °C with either 5 M CFSE in CTL medium (CFSE^high^ cells) or 0.5 M CFSE in CTL medium (CFSE^low^ cells) and washed twice with HBSS. CFSE^high^ cells were pulsed with MHC class-I restricted HA peptide (1 μg/ml) or MHC class-II restricted HA peptide (100 μg/ml) in CTL medium for 1 h at 37 °C. CFSE^low^ cells were incubated in CTL medium without peptide, serving as an internal control. CFSE labeled cells were then washed twice with HBSS. A mixture of 2.5 × 10^6^ CFSE^high^ HA peptide-pulsed cells and 2.5 × 10^6^ CFSE^low^ non-pulsed cells were injected intravenously through the tail vein 10 hours prior to the time points indicated in the text of results. After 10 hours of *in vivo* incubation, splenocytes were harvested and single cell suspensions were analyzed for detection and quantification of CFSE-labeled cells. Loss of CFSE^high^ HA peptide-pulsed cells was converted to percentage of specific killing [(CFSE^low^ − CFSE^high^)/CFSE^low^].

### Viral receptors on the lung cell surface

Lungs were minced and treated with collagenase for 30 minutes at 37 °C. Red blood cells were lysed and single cell suspensions were surface stained with *Sambucus nigra* lectin (SNA) for α-2,6 sialic acids, *Maackia amurensis* (MAA-II) for α-2,3 sialic acids and peanut agglutinin (PNA) for desialylated cells. Stained cells were acquired through FACSCalibur and analyzed by CellQuest Pro software.

### Statistical analyses

Data are represented as means ± S.D. We used Graph Pad Prism version 5 for Student’s t-test analyses. We considered all P values >0.05 not to be significant.

## Additional Information

**How to cite this article**: Dutta, A. *et al*. Sterilizing immunity to influenza virus infection requires local antigen-specific T cell response in the lungs. *Sci. Rep.* **6**, 32973; doi: 10.1038/srep32973 (2016).
